# Pathological complete response, category change, and prognostic significance of HER2-low breast cancer receiving neoadjuvant treatment: a multicenter analysis of 2489 cases

**DOI:** 10.1038/s41416-023-02403-x

**Published:** 2023-08-21

**Authors:** Siji Zhu, Yujie Lu, Xiaochun Fei, Kunwei Shen, Xiaosong Chen

**Affiliations:** 1 Department of General Surgery, Comprehensive Breast Health Center, Ruijin Hospital, Shanghai Jiao Tong University School of Medicine, Shanghai, 200025 China; 2 Department of Pathology, Ruijin Hospital, Shanghai Jiao Tong University School of Medicine, Shanghai, 200025 China

**Keywords:** Breast cancer, Chemotherapy, Targeted therapies

## Abstract

**Background:**

HER2-low breast cancers (BC) show a good response to novel anti-HER2 antibody-drug conjugates (ADCs) in advanced setting. Nevertheless, little is known about the response, category change, and prognosis of HER2-low BC receiving neoadjuvant treatment (NAT).

**Methods:**

Consecutive invasive BC patients who underwent ≥ 4 cycles of NAT and surgery from January 2009 to December 2020 were retrospectively reviewed. HER2-low was defined as IHC 1+ or 2+ and FISH negative. Concordance rates of HER2 and other biomarkers were analyzed by Kappa test. Kaplan–Meier analysis and Cox regression were used to assess the recurrence-free interval (RFI) and overall survival (OS).

**Results:**

A total of 2489 patients were included, of whom 1023 (41.1%) had HER2-low tumors. HER2-low patients had a higher ER positivity rate than HER2-0 patients (78.5% vs. 63.6%, *P* < 0.001), and a similar breast pathological complete response (pCR) rate (20.6% vs. 21.8%, *P* = 0.617). Among non-pCR cases, 39.5% of HER2-0 tumors changed to HER2-low, and 14.3% of HER2-low tumors changed to HER2-0 after NAT. Low concordance rates of HER2-low status were found in both ER-positive (Kappa = 0.368) and ER-negative (Kappa = 0.444) patients. Primary HER2-low patients had a significantly better RFI than HER2-0 patients (*P* = 0.014), especially among ER-positive subset (*P* = 0.016). Moreover, HER2-low category change was associated with RFI in ER-positive subset (adjusted *P* = 0.043).

**Conclusions:**

Compared with HER2-0 patients, HER2-low patients had a high proportion of ER-positive tumor and a similar pCR rate, which were related with better prognosis, especially in residual cases after NAT. A remarkable instability of HER2-low status was found between the primary and residual tumor, indicating re-testing HER2 status after NAT in the new era of anti-HER2 ADCs therapy.

## Introduction

Breast cancer (BC) is a highly heterogeneous disease and can be divided into four or five different subtypes according to hormone receptor (HR) and human epidermal growth factor 2 (HER2) status [1, 2]. In recent decades, a wide variety of anti-HER2 drugs have been developed for targeting tumors with HER2 amplification/overexpression [3], which has dramatically improved the prognosis of HER2-positive BC [4, 5]. HER2-negative BCs, however, show no response to most conventional anti-HER2 drugs [6, 7]. However, recent findings have challenged this dogma. Approximately half of BCs show low to moderate levels of HER2 immunohistochemical (IHC) expression (IHC 1+ or 2+ in the absence of HER2 gene amplification by in situ hybridization (ISH)) and are thus classified as HER2-low BCs [8]. Early-phase clinical trials have reported HER2-low tumors with a surprising response to novel anti-HER2 antibody-drug conjugates (ADCs), such as trastuzumab deruxtecan (T-DXd) and trastuzumab duocarmazine [9–11]. Two phase 3 trials have currently evaluated the efficacy of T-DXd in HER2-low BCs (DESTINY-Breast04 and DESTINY-Breast06), and the DESTINY-Breast04 study has reported exciting positive results in the metastatic setting [12]. Continuous attempts to transfer this experimental scenario to the early setting are in progress. Additionally, plenty of attempts are also ongoing towards defining the specific biological characteristics of HER2-low BCs [13–17]. At present, the current algorithm for HER2 testing can distinguish HER2-low tumors from HER2-0 (IHC 0) tumors [18], but the clinical implications of HER2-low expression remain debatable. It is not clear whether HER2-low tumors represent distinct subtypes of breast cancer with unique biological characteristics, and moreover, whether HER2-low early-stage breast cancer patients have different clinicopathological characteristics with different survival outcomes compared to HER2-0 patients.

For early-stage breast cancer patients, neoadjuvant treatment (NAT) currently represents a preferred option. Over past decades, neoadjuvant treatment has demonstrated an ability of in vivo evaluation of treatment sensitivity, downstaging of primary tumor and the possibility of tailoring post-neoadjuvant approaches [19–21]. In the pre-ADCs era, several studies have reported the discordance of HER2 status as a dichotomous variable from baseline biopsy to residual disease in patients undergoing neoadjuvant treatment, which was possibly due to tumor heterogeneity, assessment technological difference or evolution driven by treatment selection [22–24]. However, those previous studies did not include the HER2-low category in their evaluations. Recently, our research team reported a high discordance rate of HER2 low status between paired core needle biopsy (CNB) and surgical excision sample (SES) in early-stage breast cancer patients not receiving NAT, which emphasized the importance of exploring the inconsistency of HER2-low expression from primary breast cancer to matched residual disease in neoadjuvant setting [25]. Furthermore, to date, little is known about the treatment response of early-stage HER2-low breast cancers to NAT. It is not clear whether HER2-low BCs have different pathological complete response (pCR) after neoadjuvant chemotherapy, and whether pCR could be used as a surrogate marker of prognosis for this entity. Therefore, clarifying whether HER2-low BCs have different sensitivity to neoadjuvant chemotherapy with distinct behavior and prognosis is needed. Better understanding of those characteristics of HER2-low BCs will be essential for developing future BC therapies.

Hence, in the present work, we evaluated a large cohort of early breast cancer patients undergoing neoadjuvant chemotherapy to investigate the biological characteristics and pathological complete response to neoadjuvant treatment of HER2-low breast cancer patients, to explore the inconsistency of HER2-low expression from primary breast cancer to matched residual disease, and moreover, to evaluate the influence of HER2-low expression on prognosis.

## Patients and methods

### Study population

**Fig. 1:**
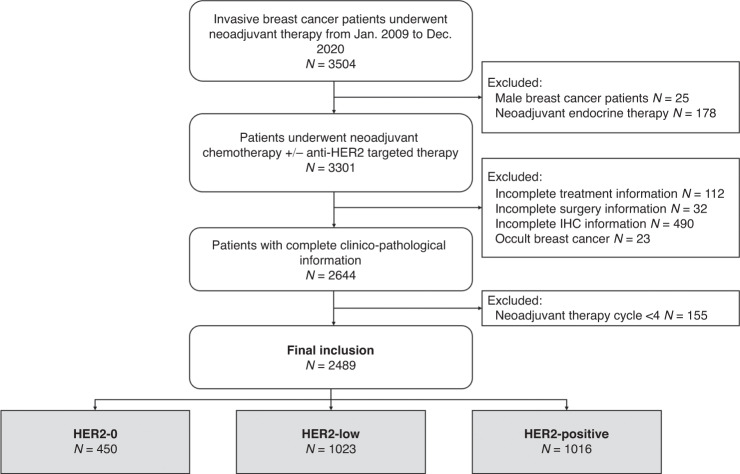
Identification of study population. HER2 human epidermal growth factor receptor-2, NAHT neoadjuvant anti-HER2 therapy, pCR pathological complete response.

Early-stage patients receiving NAT between January 2009 and December 2020 were retrospectively included. All data were retrieved from the Shanghai Jiao Tong University Breast Cancer Database (SJTU-BCDB), which included more than 70,000 breast cancer cases from 40 medical centers in China. The inclusion criteria were as follows: (a) female sex; (b) receiving ≥4 cycles of neoadjuvant chemotherapy with or without neoadjuvant anti-HER2 therapy; (c) invasive breast cancer diagnosed by core needle biopsy before NAT; and (d) complete histopathological data and clinical information before/after NAT. Male patients, occult breast cancer patients without breast lesions, patients with unknown NAT regimens or receiving neoadjuvant endocrine therapy alone, patients without accurate pathological data or surgical information, and patients who received <4 cycles of NAT were excluded (Fig. 1). This study was conducted in accordance with the Declaration of Helsinki, and approved by the independent Ethical Committees (IEC) of Ruijin Hospital. Given the anonymized nature of the data, the requirement for informed consent was waived by the IEC of Ruijin Hospital.

### Clinical and pathological assessment

Clinical tumor and nodal status before NAT were determined through physical examination (PE) or diagnostic ultrasound. Clinical breast cancer TNM staging was based on the 8^th^ American Joint Committee on Cancer (AJCC) staging manual [26]. Diagnosis of breast cancer was made by pretherapeutic ultrasound-guided core biopsies.

Pathological and IHC evaluation of tumors was accomplished by at least two independent experienced pathologists of local laboratories with standard techniques and antibodies according to American Society of Clinical Oncology/College of American Pathologists (ASCO/CAP) recommendation. The applied criteria for estrogen receptor (ER) and progesterone receptor (PR) IHC evaluation followed the latest ASCO/CAP guidelines, in which the positivity of ER/PR was defined as no less than 1% of the invasive tumor cells stained positive by IHC [27]. Ki67 assessment was followed the standard procedure recommended by the International Ki67 in Breast Cancer Working Group [28].

The algorithms for HER2 testing were performed according to the ASCO/CAP guidelines [18]. Given the definition of HER2 positivity by ASCO/CAP recommendation revised, all cases diagnosed before 2014 were reviewed by expert pathologists to comply with the currently adopted 10% cut-off. HER2 protein expression was first determined by IHC, and stratified as IHC 0, IHC 1 + , IHC 2 + , and IHC 3 + . IHC 2+ samples were subsequently subjected to carry an extra fluorescence in situ hybridization (FISH) test using a *HER2/CEP17* dual probe to detect the HER2 gene amplification. Based on the outcomes of IHC and FISH, HER2 status was classified as: HER2-positive if IHC 3 + , or IHC 2+ and FISH-positive (FISH + , dual-probe *HER2/CEP17* ratio of ≥ 2.0 with an average *HER2* copy number ≥ 4.0 signals/cell, or dual-probe *HER2/CEP17* ratio of <2.0 with an average *HER2* copy number ≥6.0 signals/cell). HER2-low was defined as IHC 1 + , or IHC2+ and FISH-negative (FISH-, dual-probe *HER2/CEP17* ratio of <2.0 with an average *HER2* copy number <6.0 signals/cell, or dual-probe *HER2/CEP17* ratio of ≥2.0 with an average *HER2* copy number <4.0 signals/cell) and HER2-0 referred to HER2 IHC 0 [29].

HER2-loss was defined as HER2 positive change to HER2-low and/or HER2-0 in non-pCR patients after NAC. HER2-negative non-pCR patients were divided into four groups according to HER2 status category change from CNB sample to residual tumor: Group A: HER2- 0 → 0; Group B: HER2-0→ low; Group C: HER2-low→ 0; Group D: HER2-low→ low.

### Outcomes

Breast pCR was defined as the absence of invasive tumor in the breast (ypT0/is). The CPS-EG score was calculated based on clinical stage, pathological stage, grade, and ER status [30]. According to the STEEP criteria [31], recurrence-free interval (RFI) (defined as the time from the date of surgery to the first relapse of tumor including ipsilateral, local/regional or distant recurrence, and death from breast cancer cause) and overall survival (OS) (defined as the time from the date of surgery untill death from any cause) were calculated. The last follow-up was completed by June of 2022.

### Statistical analysis

Statistical analysis and image construction were performed using IBM SPSS version 25 (SPSS, Inc., Chicago, IL) and GraphPad Prism version 8.0 (GraphPad Software, CA, USA), and a two-sided *P* value of <0.05 was considered statistically significant.

The chi-squared test (χ^2^) was used to compare categorical variables across groups. The concordance rates of HER2, ER, PR, and Ki67 status from primary breast cancer to residual disease after neoadjuvant treatment were analyzed by using the Kappa test, and Kappa value < 0.2, 0.2-0.4, 0.4-0.6 and > 0.6 were considered as poor, fair, moderate and good agreement, respectively. The category change of HER2 expression was graphically reported by building Sankey diagrams. Spearman correlation coefficient was used to analyze the correlation between ER expression level and HER2 category change. Kaplan–Meier curves were conducted to compare clinical outcomes according to pCR status, primary HER2 status and HER2 status change. Cox-regression model was applied to the calculate adjusted *P* value.

## Results

### Patient cohorts and clinicopathologic characteristics

A total of 2489 early-stage breast cancer patients from 32 centers (Supplementary Fig. S1) who underwent neoadjuvant chemotherapy were included: 450 patients with HER2-0 tumors, 1023 with HER2-low tumors, and 1016 with HER2-positive tumors (Fig. 1). Baseline clinical features and pathological characteristics based on CNB samples are shown in Table 1. In the overall population, the median age was 50.0 (range 21-86) years old. Invasive ductal carcinoma (IDC) was diagnosed in 94.5% of patients, and grade III tumors were found in 24.3% of patients. A total of 155 (6.2%) patients were classified as Stage I disease. Stage II and III disease were found in 1591 (63.9%) and 737 (29.6%) patients, respectively.

**Table 1:** Main clinic-pathologic characteristics.

Characteristics	Overall *N* = 2489 (%)	HER2-0^a^ *N* = 450 (%)	HER2-low^a^ *N* = 1023(%)	HER2-positive^a^ *N* = 1016 (%)	*P* value
Age, years (median, range)	50 (21–86)	49 (26–77)	50 (22–86)	51 (21–77)	0.014
<50	1183 (47.5)	226 (50.2)	510 (49.9)	457 (44.0)	
≥50	1306 (52.5)	224 (49.8)	513 (50.1)	569 (56.0)	
Menstruation					0.015
Pre/peri-menopausal	1327 (53.3)	245 (54.4)	575 (56.2)	507 (49.9)	
Post-menopausal	1162 (46.7)	205 (45.6)	448 (43.8)	509 (50.1)	
Histology					0.181
IDC	2353 (94.5)	427 (94.9)	957 (93.5)	909 (95.4)	
non-IDC	136 (5.5)	23 (5.1)	66 (6.5)	47 (4.6)	
Grade					0.005
I	39 (1.6)	15 (3.3)	13 (1.3)	11 (1.1)	
II	1064 (42.8)	181 (40.2)	472 (46.1)	411 (40.5)	
III	605 (24.3)	128 (28.4)	239 (23.4)	238 (23.4)	
NA	781 (31.4)	126 (28.0)	299 (29.2)	356 (35.0)	
Clinical TNM					<0.001
I	155 (6.2)	26 (5.8)	54 (5.3)	75 (7.4)	
II	1591 (63.9)	308 (68.4)	641 (62.7)	642 (63.2)	
III	737 (29.6)	114 (25.3)	325 (31.8)	298 (29.3)	
NA	6 (0.2)	2 (0.4)	3 (0.3)	1 (0.1)	
Primary ER					<0.001
Positive	1672 (67.2)	286 (63.6)	803 (78.5)	583 (57.4)	
Negative	817 (32.8)	164 (36.4)	220 (21.5)	433 (42.6)	
Primary PR					<0.001
Positive	1479 (59.4)	268 (59.6)	717 (70.1)	493 (48.6)	
Negative	1010 (40.6)	182 (40.4)	306 (29.9)	522 (51.4)	
Primary Ki67					<0.001
<14%	314 (12.6)	73 (16.2)	155 (15.2)	86 (8.5)	
≥14%	2145 (86.2)	370 (82.2)	860 (84.1)	915 (90.1)	
NA	30 (1.2)	7 (1.6)	8 (0.8)	15 (1.4)	
Breast Surgery					<0.001
Mastectomy	2191 (88.0)	376 (83.6)	891 (87.1)	924 (90.9)	
BCS	298 (12.0)	74 (16.4)	132 (12.9)	92 (9.1)	
Axillary Surgery					0.086
SLNB	220 (8.8)	30 (6.7)	87 (8.5)	103 (10.1)	
ALND ± SLNB	2269 (91.2)	420 (93.3)	936 (91.5)	913 (89.9)	
NAC strategy					<0.001
Anthracycline + Taxane	1670 (67.1)	342 (76.0)	822 (80.4)	506 (49.8)	
Anthracycline	161 (6.5)	41 (9.1)	77 (7.5)	43 (4.2)	
Taxane	646 (26.0)	64 (14.2)	118 (11.5)	464 (45.7)	
Others/NA	12 (0.5)	3 (0.7)	6 (0.6)	3 (0.3)	
NAC Cycle					0.450
4–6	1562 (62.8)	281 (62.4)	629 (61.5)	652 (64.2)	
>6	927 (37.2)	169 (37.6)	394 (38.5)	364 (35.8)	
Breast pCR					<0.001
No	1797 (72.2)	352 (78.2)	812 (79.4)	633 (62.3)	
Yes	692 (27.8)	98 (21.8)	211 (20.6)	383 (37.7)	

*HER2* human epidermal growth factor receptor-2, *IDC* invasive ductal carcinoma, *NA* not available, *ER* estrogen receptor, *PR* progesterone receptor, *TBCS* breast-conserving surgery, *SLNB* sentinel lymph node biopsy, *ALND* axillary lymph node dissection, *NAC* neoadjuvant chemotherapy, *pCR* pathological complete response.

^a^HER2 status is according to core needle biopsy specimen before NAC.

In terms of neoadjuvant treatment regimens, 99.5% of patients underwent anthracycline- and/or paclitaxel-based neoadjuvant chemotherapy, while 1670 (67.1%) patients were treated with anthracyclines plus taxanes. Moreover, 1562 (62.8%) patients received 4–6 cycles of NAT and 927 (37.2%) were treated with more than six cycles. Of note, 28.4% (289/1016) of HER2-positive patients received chemotherapy only without anti-HER2 targeted therapy during neoadjuvant treatment. After NAT, mastectomy and axillary lymph node dissection were carried out in 88.0% and 91.2% of patients, respectively. After surgery, patients with ER positivity in either pre- or post-neoadjuvant therapy tumor sample were recommended to have adjuvant endocrine therapy. Since all patients of current study were firstly treated before 2021 when the immune check-point inhibitors were not approved in China, none of them received immune check-point inhibitors in neoadjuvant and adjuvant setting.

### Features and pathological response of HER2-low breast cancer

According to HER2 status on CNB, the distribution of HER2 expression for all HER2-negative patients (*N* = 1473) was as follows: HER2-0 30.5%, HER2-low 69.5%. A higher ER positivity rate was observed in HER2-low cases than in HER2-0 cases (78.5% vs. 63.6%, *P* < 0.001), as well as PR positivity rate (70.1% vs. 59.6%, *P* < 0.001), as shown in Table 1.

There were 692 (27.8%) patients achieving breast pCR after NAT (Table 2). The breast pCR rates of ER-positive and ER-negative patients were 25.3% and 32.9%, respectively (*P* < 0.001). In addition, the pCR rate in ER-negative patients according to tumor stage, neoadjuvant regimens, and neoadjuvant therapy cycles was shown in Supplementary Fig. S2. When evaluating the breast pCR according to ER and HER2 status, multivariate analysis showed that ER status significantly influenced pCR rates of HER2-low (HR 0.47, 95% CI 0.29–0.75, adjusted *P* = 0.002) and HER2-positive (HR 0.68, 95% CI 0.47–0.99, adjusted *P* = 0.045) patients, but not in HER2-0 patients (HR 0.80, 95% CI 0.31–1.29, adjusted *P* = 0.630).

**Table 2:** pCR rates in patients stratified by primary ER and HER2 status.

	ER-positive (%)	ER-negative (%)	All (%)	Univariate *P* value	Multivariate*		
					HR	95%CI	*P* value
HER2-0	21.0	23.2	21.8	0.635	0.80	0.31–1.29	0.630
HER2-low	17.7	31.4	20.6	<0.001	0.47	0.29–0.75	0.002
HER2-positive	37.9	37.4	37.7	0.896	0.68	0.47–0.99	0.045
All	25.3	32.9	27.8	<0.001	0.60	0.45–0.78	<0.001

*Multivariate Cox regression model includes age, menstruation, cTNM stage, primary ER, primary PR, primary HER2, primary Ki67 statuses, NAC strategy, NAC cycle, histology type, and histological grade.

*HER2* human epidermal growth factor receptor-2, *HR* hazard ratio, *CI* confidence interval, *ER* estrogen receptor.

**Fig. 2:**
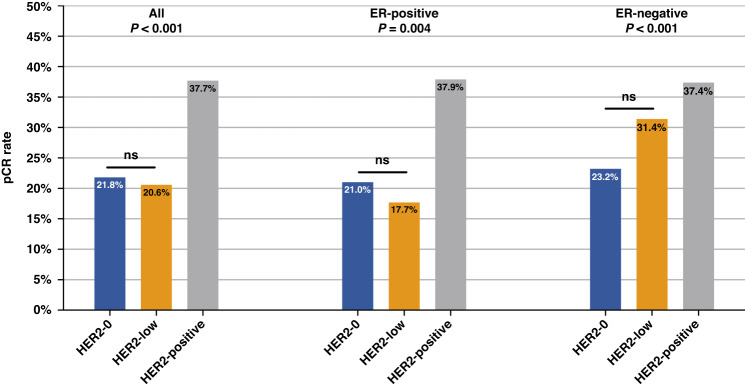
Breast pCR rates according to HER2 expression. HER2, human epidermal growth factor receptor-2; pCR, pathological complete response.

Concerning HER2 status and breast pCR, a significant association was found between HER2 expression and the probability of breast pCR. As shown in Fig. 2, breast pCR rates were 21.8%, 20.6%, and 37.7% for HER2-0, HER2-low, and HER2-positive patients, respectively (*P* < 0.001). HER2-positive patients had the highest pCR rates, while HER2-low cases had numerically low pCR rates. Among HER2-negative cases, the pCR rate was not significantly different between HER2-0 and HER2-low patients (21.8% vs. 20.6%, *P* = 0.617). Moreover, when considering each ER subset, the association between pCR rates and HER2-low expression was inconsistent: for ER-positive patients, the breast pCR rate of HER2-low cases was slightly lower than that of HER2-0 cases (17.7% vs. 21.0%, *P* = 0.218), and for ER-negative patients, the breast pCR rate of HER2-low cases was numerically higher than that of HER2-0 cases (31.4% vs. 23.2%, *P* = 0.077). Neither of the subsets reached the statistical significance.

### HER2 and other biomarkers change from CNB samples to residual disease after NAT

**Fig. 3:**
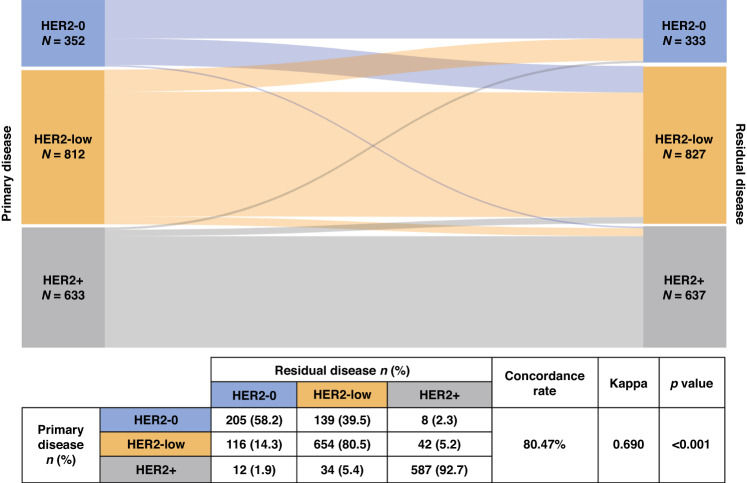
HER2 category change between primary and residual disease. HER2 human epidermal growth factor receptor-2.

The category change of HER2 expression from baseline biopsy to residual disease after NAT is shown in Fig. 3. The overall rate of HER2 discordance was 19.53% (Kappa = 0.690, *P* < 0.001). In patients with HER2-0 status at CNB, 39.5% (*N* = 139) experienced a change to HER2-low status after neoadjuvant treatment, while 14.3% (*N* = 116) showed a category change in the opposite direction. HER2-positive tumors in the CNB sample showed a high stability among three groups, with 7.3% (*N* = 46) of patients exhibiting HER2-loss, either change to HER2-0 (*N* = 12) or HER2-low (*N* = 34). Uni- and multi-variate analysis showed that ER status (OR 0.46, 95% CI 0.24–0.90, adjusted *P* = 0.024) and using anti-HER2 targeted therapy (OR 0.37, 95% CI 0.18–0.79, adjusted *P* = 0.010) during NAC were independent factors to HER2-loss (Supplementary Fig. S3). Similar result was found when we evaluated the category change from HER2-positive to HER2-low (ER status: OR: 0.33, 95 CI 0.14–0.78, adjusted *P* = 0.012; usage of anti-HER2 therapy: OR: 0.26, 95 CI 0.10–0.68, adjusted *P* = 0.006). When further dividing the patients into four subgroups according to the IHC and FISH results: HER2 0, HER2 1 + , HER2 2 + /FISH-, and HER2 3+ or 2 + /FISH + . The overall discordance rate of HER2 expression was 31.22% (Kappa = 0.575, *P* < 0.001, Supplementary Fig. S4).

When focusing on HER2-negative cases with residual disease after NAT, we further divided those patients into two subgroups according to ER status in CNB samples (Supplementary Fig. S5). Among ER-positive patients, 99/226 (43.8%) HER2-0 cases changed to HER2-low, and 85/661 (12.9%) HER2-low cases changed to HER2-0 after neoadjuvant treatment (Kappa = 0.368, *P* < 0.001). Among ER-negative patients, 40/126 (31.2%) cases moved from HER2-0 to HER2-low status, and 31/151 (20.5%) patients exhibited a change from HER2-low to HER2-0 status after NAT (Kappa = 0.444, *P* < 0.001). The detail of HER2 category change according to ER expression level is shown in Supplementary Fig. S6, with a Spearman correlation index of 0.241, which showed a weak correlation between ER expression level and HER2 category change. In addition, the HER2-low proportion and HER2 category change across different centers and different enroll periods were shown in Supplementary Tables S1 and S2. No significant difference in HER2-low proportion and HER2 category change was observed across different centers and different enroll periods.

With regard to other biomarkers (Supplementary Table S3), high concordance rates were found with good agreements in ER (concordance rate 91.90%, Kappa = 0.812, *P* < 0.001), PR (concordance rate 85.30%, Kappa = 0.697, *P* < 0.001) and HER2 as dichotomous variable (concordance rate 94.70%, Kappa = 0.883, *P* < 0.001) evaluation between CNB and matched residual samples. Only the Ki67 category (taking 14% as a cutoff) showed a large variation with fair agreement after neoadjuvant therapy (concordance rate 67.30%, Kappa = 0.254, *P* < 0.001).

### Survival analysis of HER2-low patients according to pCR status

**Fig. 4:**
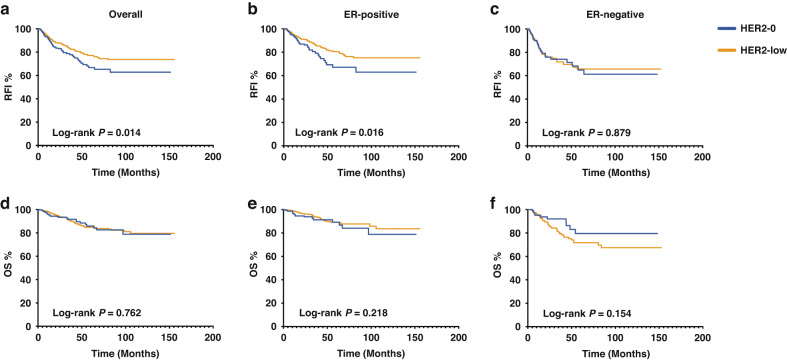
Survival outcome according to primary HER2-low expression. RFI and OS according to primary HER2-low expression of (**a**, **d**) all, (**b**, **e**) ER-positive, and (**C**, **F**) ER-negative, HER2-negative patients. RFI recurrence free interval, OS overall survival, HER2 human epidermal growth factor receptor-2, ER estrogen receptor.

Survival analysis was conducted to assess RFI and OS. At a median follow-up time of 24.4 (range 5.5–155.6) months, 154 patients were dead and disease recurrence events were found in 308 patients. In whole population, the uni- and multi-variate analysis showed that HER2 status is an independent prognostic factor after adjusting to hormone receptor status and others factors in terms of RFI and OS, and HER2-low patients had a superior RFI compared to HER2-0 patients (Hazard ratio=0.66, 95% CI 0.49–0.89, *P* = 0.007) (Supplementary Table S4, S5). Focusing on the HER2-negative population, as shown in Fig. 4a, HER2-low patients showed a significantly better RFI than HER2-0 patients (the estimated 5-year RFI: 66.7% vs. 77.1%; *P* = 0.014). When further dividing patients according to ER status, there was a significantly superior RFI of ER-positive/HER2-low patients than that of ER-positive/HER2-0 patients (*P* = 0.016) (Fig. 4b). However, in the ER-negative subset, the advantage of RFI was not observed in HER2-low patients *(P* = 0.879) (Fig. 4c). Regarding OS, no significant differences were found between HER2-0 and HER2-low patients among all HER2-negative (Fig. 4d, *P* = 0.762), ER-positive/HER2-negative (Fig. 4e, *P* = 0.218) or ER-negative/HER2-negative patients (Fig. 4f, *P* = 0.154). Annual relapse and death risk cures are shown in Supplementary Fig. S7. Relapse cures showed that HER2-low group had a similar but lower early-recurrence risk compared to HER2-0 group, and the cures of two groups crossed after 8 years.

**Fig. 5:**
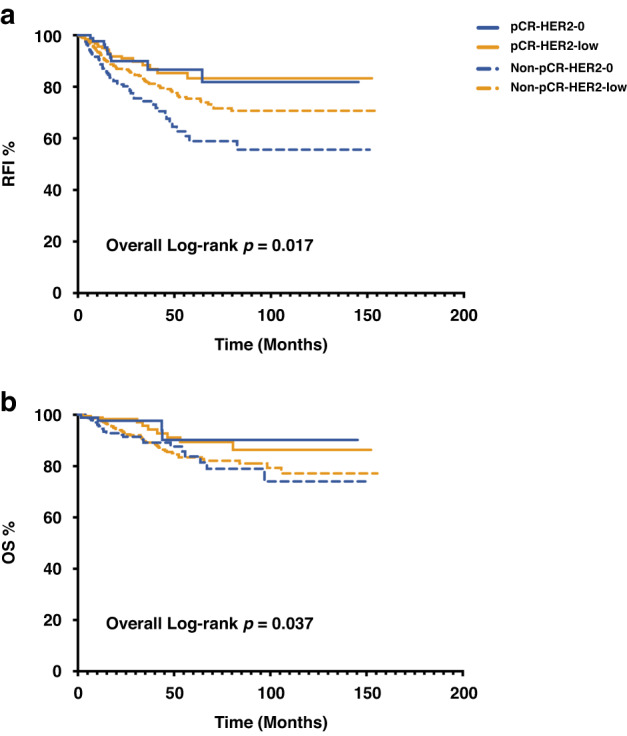
Survival outcome according to pCR status in primary HER2-negative patients. RFI (**a**) and OS (**b**) according to pCR status in primary HER2-negative patients. Log-rank *P* value for non-pCR-HER2-0 VS. non-pCR-HER2-low patients: RFI, *P* = 0.004; OS, *P* = 0.532. Log-rank *P* value for pCR-HER2-0 VS. pCR-HER2-low patients: RFI, *P* = 0.919; OS, *P* = 0.864. RFI recurrence free interval, OS overall survival, pCR pathological complete response, HER2 human epidermal growth factor receptor-2.

When evaluating the prognostic impact of primary HER2-low expression according to pCR status, we divided all those HER2-negative patients into pCR and non-pCR groups. Among the pCR group, there was no significant difference in RFI (*P* = 0.919) or OS (*P* = 0.864) between HER2-0 and HER2-low when achieving pCR. Regarding non-pCR patients, the estimated 5-year RFI rates for HER2-0 and HER2-low, were 58.9% and 75.3%, respectively (*P* = 0.004) (Fig. 5a). In contrast, no significant difference in OS was observed between HER2-0 and HER2-low non-pCR patients (*P* = 0.532) (Fig. 5b). Further dividing the non-pCR patients according to ER positivity, the advantage of RFI was observed in the ER-positive subset (*P* = 0.007) (Supplementary Fig. S8A), but not in the ER-negative subset (*P* = 0.987) (Supplementary Fig. S8B). In terms of OS, similar to the result of the whole population, no significant difference was observed in either the ER-positive (*P* = 0.121) or the ER-negative (*P* = 0.131) groups (Supplementary Fig. S8C, D).

To further identify the prognostic impact of HER2 category change from baseline biopsy to residual disease in HER2-negative cases, we evaluated the RFI and OS according to HER2-low status change. There was a significant difference observed for RFI (HER2-0 → 0 vs. HER2-0→low vs. HER2-low→0 vs. HER2-low→low, *P* = 0.029) (Supplementary Fig. S9A). The difference did not reach statistical significance when adjusted for age, menstruation, stage, PR, Ki67, and grade. When dividing patients according to ER status, HER2 category change was found to be associated with RFI among ER-positive group after adjustment (*P* = 0.043) (Supplementary Fig. S9B). Among those ER-positive patients, HER2-0→low patients had the lowest RFI, while HER2-low→low patients had the highest RFI. Similarly, regarding OS, a significant difference in the four groups was not observed in the whole population but was observed among those ER-positive patients (adjusted *P* = 0.020) (Supplementary Fig. S9D, E).

Furthermore, the CPS-EG scores were calculated in HER2-negative patients. The CPS-EG score distribution and category according to HER2 expression and HER2 category change is shown in Supplementary Fig. S10. No statistical significance was observed for CPS-EG score distribution when comparing HER2-low to HER2-0 (*P* = 0.385, Supplementary Fig. S10A) and among different HER2 category change groups (HER2-0 → 0 vs. HER2-0→low vs. HER2-low→0 vs. HER2-low→low, *P* = 0.302, Supplementary Fig. S10C). Concerning the category of CPS-EG, 46.3% of HER2-0 and 44.0% of HER2-low patients had an estimated high risk of relapse based on a CPS-EG score ≥3, which did not reach statistical significance (*P* = 0.744, Supplementary Fig. S10B). Similarly, no statistical significance of CPS-EG score ≥3 classes was observed among different HER2 category change groups (HER2-0 → 0 vs. HER2-0→low vs. HER2-low→0 vs. HER2-low→low, *P* = 0.131, Supplementary Fig. S10D).

## Discussion

HER2-low breast cancer is gaining attention not only because of its potential clinical benefits from novel ADCs but also because of its possibly unique biological characteristics compared with HER2-0 and HER2-positive BCs (**A summary of current studies cornering HER2-low BCs in NACT shown in** Supplementary Table S6**)**. To our knowledge, our study is one of the largest multicenter cohort analyses focused on the pCR of HER2-low BC to neoadjuvant chemotherapy, the inconsistency of HER2-low expression between the primary disease and matched residual disease samples, and the biologic and prognostic significance of HER2-low expression in BC patients undergoing neoadjuvant chemotherapy. We demonstrated a high proportion of ER positive tumor and a similar pCR rate of HER2-low BC compared with HER2-0 BC, which was related to a better prognosis in patients with residual disease treated with NAT. A high discordance rate of 19.53% of HER2-low status was found between primary tumor and residual disease, indicating the importance of re-testing HER2 status after NAT in the new era of anti-HER2 ADC therapy.

Concerning the biological characteristics of HER2-low BCs, there is an ongoing debate on whether HER2-low BC is a distinct entity with distinct characteristics, but data on this subject are still lacking. In our study, we found that the largest numerically and most clinically relevant clinicopathologic differences between HER2-low and HER2-0 tumors were in ER and PR expressions. In our cohort, HER2-low patients had significantly higher ER and/or PR positivity rates than HER2-0 patients. This finding was consistent with several other studies. Recently, Tarantino et al. and Denkert et al. both found that ER and HER2-low had a positive association, with the rate of HER2-low tumors increasing progressively with increased ER expression [32]. These results were also supported by gene expression analyses from Schettini et al., which revealed a higher rate of luminal-like tumors among HER2-low tumors and a higher rate of basal-like tumors among HER2-0 tumors by PAM50-based intrinsic subtyping [13].

In addition, by using the breast pCR definition, we observed a similar pCR rates in patients with the HER2-low phenotype as compared to HER2-0. When restricting the analysis to patients with ER-positive or ER-negative tumors, we did not observe any significant difference in the pathological complete response rate between tumors with HER2-0 versus HER2-low status. This observation appears close to recent reports. In a retrospective report of a cohort of 446 patients [33], HER2-low patients exhibited a lower pCR than HER2-0 tumors, but when assessing pCR rates separately in HR-positive/HER2-negative and TN subgroups, the association between HER2 expression and pCR was no longer significant. Denkert et al. also found that HER2-low tumors had a significantly lower pCR rate than HER2-0 tumors, but subset analyses showed that the difference was only significant in the HR-positive subgroup, but not in the HR-negative subgroup [34]. All these results suggest that in the HER2-negative cohort, the major determinant of chemosensitivity was HR status rather than HER2 expression, and HR positivity may be a confounder in comparisons between HER2-low and HER2-0 tumors. Moreover, we noticed an interesting phenomenon that ER positivity is correlated with a lower pCR rate only in the HER2-low subgroup but not in the HER2-0 population. The possible explanations might include: low-level HER2 expression in ER-positive patients can possibly induce treatment resistance due to the crosstalk between HER2 and ER pathway, and the role of HER2 low expression may interact with ER-positive breast cancer but not in ER-negative patients, as estrogen has little effect in the ER-negative group. Also, this interact would not exist in the HER-0 patients due to the lack of HER2 expression. Indeed, as expected, we observed the highest pCR rate in HER2-positive tumors. In addition, the pCR rates of ER-negative patients in our cohort was relatively low, with 27.7% for TNBC and 37.4% for ER-negative/HER2-positive patients. The main reasons for these results might include: there were nearly 35% of patients with LABC enrolled in our study, whom with large tumor burden were less likely to achieve pCR. Also, in our cohort, few of TNBC patients received nab-paclitaxel, none of them received anti-PD-1/PD-L1 inhibitors, and about 30% of HER2-positive patients did not receive trastuzumab or other anti-HER2 drugs. The mixture of neoadjuvant regimens may influence the achievement of pCR, as shown in in Supplementary Fig. S2.

Furthermore, we explored the inconsistency of HER2-low expression between the baseline tumor and residual disease in patients undergoing neoadjuvant chemotherapy, by adopting a HER2-based three-tier algorithm. Concerning the category change of HER2-low for BC, our team recently reported a high discordant rate (23.13%) of HER2-low status between paired CNB and SES samples in treatment-naïve early breast cancer patients [25]. In addition, Miglietta et al. also evaluated the discordance of HER2-low expression from primary to recurrent breast cancer, which showed an overall rate of 38.0% for HER2-low discordance [35]. In the present study, we observed a 19.53% overall rate of HER2 discordance between baseline biopsies and residual disease samples, due to HER2-low cases changing either from or to HER2-0 expression. This finding from the present work solidifies the remarkable instability of HER2-low expression in the early-stage breast cancer setting. In contrast, HER2-positive breast cancer shows a good concordance after NAT with only 46 of 633 patients converted to the HER2-0 or HER2-low phenotype on residual disease. We also evaluated of HR and HER2 status as dichotomous variables from baseline biopsy to residual disease after NAT, observing the discordance rates of 8.1% for ER, 14.7% for PR, and 5.3% for HER2, thus further consolidating the value of biomarker status re-evaluation after NAT in cases of non-pCR.

Regarding this result, first, this susceptibility of HER2-low expression to change after the exposure to NAT adds to available evidence suggesting HR and/or HER2 status discordance from primary tumor to residual disease after neoadjuvant treatment as a relatively frequent phenomenon [22–24, 36, 37]. We identified nearly 40% of patients with HER2-0 phenotype at baseline showing a category change to HER2-low expression after NAT. This result suggested that the evaluation of HER2 expression on residual disease may allow the access to potentially effective novel treatment strategies in a nonnegligible proportion of patients who would otherwise be excluded based on the primary tumor phenotype. In this context, our findings emphasize the importance of reprofiling the tumor on residual disease, on the other hand, they support the inclusion of the HER2-low category in this evaluation. Indeed, our study anticipated the forthcoming and imperative need to identify the proper patients who may obtain access to novel HER2-targeted treatment, as well as properly selecting those who may benefit from these novel strategies in the new ADCs era [38].

The more important question is why HER2-low status changes occur between CNB samples and residual tumors. First, it should be noted that in our present cohort, all patients underwent chemotherapy, thus precluding the possibility of uncovering whether the category change of HER2-low status reflects a genuine shift as a consequence of chemotherapy exposure. In this context, the discordant rate of HER2-low expression between CNB samples and treatment-naïve surgical specimens was important, which we had reported in our other recent study [25]. Second, the intratumoral heterogeneity of HER2-low tumor may also contribute to such analytical variability, which has been described in several studies. Recently, three studies showed that, by using the PAM50, MammaPrint, and BluePrint tests, despite the majority of luminal subtypes, HER2-low cancers also contain a small portion of the HER2-enriched subtype (3.5-3.6%) and basal-like(13.3–17.7%) subtypes [39]. These results indicated that HER2-low BC diagnosed by CNB before NAT is a mixture of several tumor clusters, thus resulting in HER2-low status change after NAT. Moreover, the technical aspects of HER2 testing methods are likely a major reason for the HER2 discrepancy. Technical variations and preanalytical factors, such as the poor agreement of HER2 antibodies used in IHC assays, may cause the poor reproducibility of HER2-low diagnosis [40, 41]. Besides, according to the current guidelines, the FISH/IHC test is a reliable tool to differentiate HER2-positive tumors from HER2-negative tumors [42], but its actual sensitivity, as a semi-quantitative assay, in detecting low levels of HER2 expression may be questionable. Combined with recent data showing the activity of anti-HER2 ADCs in HER2-0 BC, such as the DAISY phase 2 trial [43], in which T-DXd achieved an objective response rate of 30% for metastatic HER2-0 BC, it might be worth investigating whether novel quantitative HER2 assays, including those based on mRNA expression and proteomics analyses, may better refine evaluation of HER2 expression and treatment selection for novel ADCs [44].

On the other hand, in our research, there are 7.3% of HER2-positive patients failing to achieve pCR changed to HER2-low or HER2-0 after neoadjuvant treatment, and therefore lost HER2 positivity. Our result showed HER2-positive patients with ER-positive tumors and using anti-HER2 targeted drug during NAC were more likely to exhibit HER2-loss. Although the results from the KATHERINE trial, which established TDM1 as the standard post-NAT strategy in HER2-positive patients with non-pCR after NAT, revealed that patients with HER2-loss at surgery still could receive benefit from TDM1 over trastuzumab [45]. However, it is reasonable to speculate that the by-stander effect of novel anti-HER2 ADCs (e.g. T-DXd) may offer a greater advantage to those patients converting from HER2-positive to HER2-low in the residual disease after neoadjuvant therapy, which needs further validation.

Finally, focusing on HER2-low patients, we conducted an exploratory survival analysis. According to our results, HER2-low patients as a new distinct subset had an independent prognostic feature from hormone receptor status, with a better prognosis than HER2-0 patients. For pCR patients, the prognosis is very good irrespective of the HER2­0 versus HER2­low status, which reveals that pCR retains a prognostic role in HER2-low BC. This result emphasized the importance of new therapies targeting low­HER2­expressing cells to achieve a higher pCR rate and to improve the prognosis of this subgroup of patients, for example, T-DXd in the TRIO-US B-12 TALENT trial [46]. In patients with residual disease after NAT, the differences in survival between patients with HER2­0 and HER2­low tumors are relevant, especially in the ER-positive subset. Our data showed a lower early recurrence risk of HER2-low patients, especially among the ER-positive group. All these results fuel the disputable uncertainty regarding the prognosis of HER2-low early breast cancer [8, 32–34]. Moreover, among HER2-negative BC patients, HER2-low status inconsistency between baseline biopsy and residual disease after neoadjuvant chemotherapy retained its prognostic role among ER-positive patients. These data stress the notion of the most relevant implication of retesting HER2 expression on residual disease by also including the HER2-low category, especially in ER-positive patients. In addition, our evaluation of the CPS-EG score enhanced our pCR result, and there was no significant difference between patients with HER2-0 and HER2-low tumors regarding the response to neoadjuvant chemotherapy.

Our work has several strengths. To our knowledge, our study represents one of the largest cohorts of early-stage breast cancer patients undergoing neoadjuvant chemotherapy to evaluate HER2-low category change from primary tumor to residual disease. And our study included patients from a multi-center database involving 32 hospitals to provide real-world evidence with great credibility of our research. The main limitation of the current study is that the retrospective nature of our study may lead to an unavoidable diagnosis and selection mistakes, and central HER2 expression revision of all cases was not planned. Another limitation is represented by the heterogeneity of NAT.

In conclusion, our study presented a comprehensive perspective on HER2-low tumor and demonstrated the clinic-pathological characteristics of this newly raised subgroup of breast cancer in a large cohort of early-stage breast cancer patients undergoing neoadjuvant chemotherapy. Among HER2-negative tumors, HER2-low status and ER expression were positively associated. Compared with HER2-0 BC, HER2-low tumors had a similar pCR rate after neoadjuvant chemotherapy. Our study indicated that HER2-low breast cancer is a new distinct prognostic subset independent from hormone receptor status, with a better prognosis than HER2-0 patients, especially among those non-pCR patients. We revealed a remarkable instability of HER2 expression from primary breast cancer to residual disease, which indicating that regular retesting of HER2-low status in residual disease should be performed to guide further clinical anti-HER2 ADC therapy.

### Supplementary information

Supplementary Table

Supplementary Figure

## Data Availability

The data analyzed in the current study are available from the corresponding authors on reasonable request.
