# Supplementary material for: Pathological complete response, category change, and prognostic significance of HER2-low breast cancer receiving neoadjuvant treatment: a multicenter analysis of 2489 cases
Source: Br J Cancer. 2023 Aug 21;129(8):1274–83. doi: 10.1038/s41416-023-02403-x (PMC10575949; doi:10.1038/s41416-023-02403-x)
Supplement: Supplementary file 1 — Supplementary Table [file 41416_2023_2403_MOESM1_ESM.docx]

**Table S1. HER2-low proportion and HER2 category change in different centers.**

| Center^*^ | N | HER2-low (N, %) | *P* value | HER2 category change^#^ (n/N, %) | *P* value | HER2 category change in HER2-negative^#^ (n/N, %) | *P* value |
| --- | --- | --- | --- | --- | --- | --- | --- |
| A | 608 | 256 (42.1) | 0.984 | 86/444 (19.4) | 0.997 | 85/350 (24.3) | 0.995 |
| B | 559 | 220 (39.4) |  | 73/383 (19.1) |  | 61/241 (25.3) |  |
| C | 481 | 212 (41.4) |  | 51/271 (18.8) |  | 40/154 (26.0) |  |
| D | 177 | 71 (40.1) |  | 27/140 (19.3) |  | 26/99 (26.3) |  |
| E | 86 | 34 (39.5) |  | 15/75 (20.0) |  | 11/34 (32.4) |  |
| F | 84 | 32 (38.1) |  | 12/67 (17.9) |  | 12/41 (29.3) |  |
| G | 60 | 24 (40.0) |  | 13/54 (24.1) |  | 8/29 (27.6) |  |
| H | 56 | 22 (39.3) |  | 12/55 (21.8) |  | 10/32 (31.3) |  |
| I | 56 | 23 (41.1) |  | 8/36 (22.2) |  | 6/23 (26.1) |  |
| J | 53 | 21 (39.6) |  | 10/48 (20.8) |  | 8/30 (26.7) |  |
| K | 50 | 20 (40.0) |  | 10/48 (20.8) |  | 9/31 (29.0) |  |
| Others^$^ | 219 | 88 (40.2) |  | 34/176 (19.3) |  | 29/100 (26.2) |  |

* Center ID: A, Ruijin Hospital, Shanghai Jiaotong University School of Medicine; B, The First People's Hospital of Foshan; C, Henan Cancer Hospital; D, Shanxi Bethune Hospital, Shanxi Academy of Medical Science; E, The First Affiliated Hospital of Fujian Medical University; F, Jiaxing Maternal and Child Health Hospital; G, Quanzhou First Hospital, Fujian; H, The International Peace Maternity & Child Health Hospital of China welfare institute; I, Zhejiang Provincial People‘s Hospital; J, The First Affiliated Hospital of Nanchang University; K, The First People's Hospital of Zunyi.

# Patient reached breast pathological complete response after neoadjuvant therapy were excluded.

$ Centers with no more than 50 patients were combined.

**Table S2. HER2-low proportion and HER2 category change according to the time of first surgery.**

| Year of Surgery | N | HER2-low (N, %) | *P* value | HER2 category change^#^ (n/N, %) | *P* value | HER2 category change in HER2-negative^#^ (n/N, %) | *P* value |
| --- | --- | --- | --- | --- | --- | --- | --- |
| 2009-2013 | 266 | 113 (42.5) | 0.889 | 43/206 (20.9) | 0.708 | 37/138 (27.8) | 0.254 |
| 2014-2018 | 1057 | 433 (41.0) |  | 172/862 (20.0) |  | 108/510 (21.2) |  |
| 2019-2020 | 1166 | 477 (40.9) |  | 136/729 (18.7) |  | 110/471 (23.4) |  |

# Patient reached breast pathological complete response after neoadjuvant therapy were excluded

**Table S3. Concordance rate of ER, PR, HER2, and Ki67 status between primary and residual disease.**

| **Primary disease** | **Residual disease** | | **Concordance rate** | **Kappa** | ***P* value** |
| --- | --- | --- | --- | --- | --- |
|  | positive | negative |  |  |  |
| **ER** |  |  | 91.90% | 0.812 | **<0.001** |
| positive | 1157 | 92 |  |  |  |
| negative | 54 | 494 |  |  |  |
| **PR** |  |  | 85.30% | 0.697 | **<0.001** |
| positive | 920 | 190 |  |  |  |
| negative | 75 | 612 |  |  |  |
| **HER2** |  |  | 94.70% | 0.883 | **<0.001** |
| positive | 587 | 46 |  |  |  |
| negative | 50 | 1114 |  |  |  |
| **Ki67, %** | < 14 | ≥ 14 | 67.30% | 0.254 | **<0.001** |
| < 14 | 208 | 56 |  |  |  |
| ≥ 14 | 525 | 989 |  |  |  |

Abbreviations: ER, estrogen receptor; PR, progesterone receptor; HER2, human epidermal growth factor receptor

**Table S4. Univariate survival analysis of RFI and OS in all patients.**

| Characteristics | *P* value | |
| --- | --- | --- |
|  | RFI | OS |
| Age, years | 0.438 | 0.889 |
| Menstruation | 0.576 | 0.639 |
| Histology | 0.142 | 0.091 |
| Grade | **<0.001** | **<0.001** |
| Clinical TNM | **<0.001** | **<0.001** |
| Primary ER | **0.002** | **<0.001** |
| Primary PR | **<0.001** | **0.001** |
| Primary HER2 | **0.002** | **0.004** |
| Primary Ki67 | 0.809 | 0.660 |
| Primary phenotype | **<0.001** | **<0.001** |
| NAC strategy | **<0.001** | **0.003** |
| NAC Cycle | 0.199 | 0.055 |

Abbreviations: HER2, human epidermal growth factor receptor-2; RFI, recurrence free interval; OS, overall survival; ER, estrogen receptor; PR, progesterone receptor; NAC, neoadjuvant chemotherapy.

**Table S5. Multivariate survival analysis of RFI and OS in all patients.**

| Characteristics | RFI | | |  | OS | | |
| --- | --- | --- | --- | --- | --- | --- | --- |
|  | HR | 95% CI | *P* value |  | HR | 95% CI | *P* value |
| Grade |  |  | **0.005** |  |  |  | **0.010** |
| I | 1.00 |  |  |  | 1.00 |  |  |
| II | 0.80 | 0.29-2.18 | 0.656 |  | 0.83 | 0.33-2.00 | 0.766 |
| III | 1.26 | 0.46-3.48 | 0.654 |  | 1.33 | 0.64-4.01 | 0.898 |
| Clinical TNM |  |  | **<0.001** |  |  |  | **<0.001** |
| I | 1.00 |  |  |  | 1.00 |  |  |
| II | 1.62 | 0.59-4.40 | 0.347 |  | 1.78 | 0.66-4.89 | 0.793 |
| III | 3.24 | 1.19-8.81 | **0.021** |  | 4.01 | 1.81-6.79 | **0.019** |
| Primary ER |  |  | **0.034** |  |  |  | **0.039** |
| Positive | 1.00 |  |  |  | 1.00 |  |  |
| Negative | 1.34 | 1.02-1.77 |  |  | 1.43 | 1.20-1.98 |  |
| Primary PR |  |  | **0.008** |  |  |  | 0.857 |
| Positive | 1.00 |  |  |  | 1.00 |  |  |
| Negative | 1.47 | 1.11-1.96 |  |  | 1.05 | 0.61-1.82 |  |
| Primary HER2 |  |  | **0.001** |  |  |  | **0.001** |
| HER2-0 | 1.00 |  |  |  | 1.00 |  |  |
| HER2-Low | 0.66 | 0.47-0.93 | **0.017** |  | 0.93 | 0.56-1.54 | 0.774 |
| HER2-Positive | 0.50 | 0.35-0.72 | **<0.001** |  | 0.40 | 0.23-0.71 | **0.002** |
| NAC strategy |  |  | 0.155 |  |  |  | **0.014** |
| Anthracycline + Taxane | 1.00 |  |  |  | 1.00 |  |  |
| Anthracycline | 1.18 | 0.71-1.96 | 0.523 |  | 1.68 | 0.88-3.23 | 0.117 |
| Taxane | 1.48 | 1.05-2.08 | **0.024** |  | 2.08 | 1.30-3.33 | **0.002** |
| Others/NA | 0.99 | 0.10-7.74 | 0.956 |  | 0.98 | 0.05-5.13 | 0.971 |

Abbreviations: HER2, human epidermal growth factor receptor-2; HR, hazard ratio; CI, confidence interval; RFI, recurrence free interval; OS, overall survival; ER, estrogen receptor; PR, progesterone receptor; NAC, neoadjuvant chemotherapy.

**Table S6. Summary of current studies concerning HER2-low BC in neoadjuvant chemotherapy.**

| **Author, year** | **Type of Study** | **Inclusion period** | **Number of patients (HER2-0/low)** | **Method of**  **HER2 assessment** | **pCR rate of NAC (HER2-0 vs. HER2-low)** | | | **Survival rate between HER2-0 and HER2-low** | | | **Category change of HER2 status after NAC** | | |
| --- | --- | --- | --- | --- | --- | --- | --- | --- | --- | --- | --- | --- | --- |
|  |  |  |  |  | **Whole population** | **ER-positive** | **ER-negative** | **Whole Population** | **ER-positive** | **ER-negative** | **Discordance rate** | **HER2-0 to HER2-low** | **HER2-low to HER2-0** |
| Denkert, 2021^1^ | Retrospective, Multi-center | 2012-2019 | 2310 (1212/1098) | Central evaluated according to ASCO/CAP recommendations at time of inclusion into clinical trials, cases diagnosed before 2014 were reviewed to comply with the currently adopted 10% cutoff of HER2 IHC 3+ | 39.2% vs. 29.0% (P=0.0002) | 23.6% vs 17.5% (P=0.024) | 48.0% vs. 50.1% (P=0.21) | 3-year DFS 76.1% vs. 83.4% (P=0.0084)    3-year OS 85.8% vs. 91.6% (P=0.0016) | 3-year DFS 79.3% vs. 82.8% (P=0.39)  3-year OS 88.4% vs. 92.3% (P=0.13) | 3-year DFS 84.3% vs. 90.2% (P=0.016)  3-year OS 82.5% vs. 87.8% (P=0.200) | NR | NR | NR |
| Leite, 2021^2^ | Retrospective; Single center | 2007-2018 | 855 (570/285) | According to the ASCO/CAP guidelines available during the different time periods | NR | 9.5% vs 13% (P=0.27) | 47% vs. 51% (P=0.64) | NR | 5-year RFS 71.7% vs. 72.1% (P=0.47)  5-year OS 83.8% vs. 89.4% (P=0.11) | 5-year RFS 70.8% vs. 75.6% (P=0.23)  5-year OS 80.3% vs. 79.1% (P=0.71) | NR | NR | NR |
| Alves, 2022^3^ | Retrospective; Single center | 2015-2020 | 72 (31/41) | According to ASCO/CAP guidelines available at the time of diagnosis | 29.0% vs. 14.6% (P=0.15) | 27.0% vs 14.0% (P=0.36) | 30.0% vs. 17.0% (P=0.67) | 2-year DFS 84% vs. 82% (P=0.97)  2-year OS 90% vs. 95% (P=0.35) | NR | NR | NR | NR | NR |
| Di Cosimo, 2022^4^ | Retrospective; Single center | 2009-2020 | 444 (109/335) | NR | 29.36% vs. 11.64% (P<0.0001) | 10.64% vs 5.51% (P=0.1889) | 43.55% vs. 38.1% (P=0.5353) | 3-year DFS 78% vs. 73% (P=0.8533) | 3-year DFS NS | 3-year DFS NS | 21.97% | 7.51% | 14.45% |
| Shao, 2022^5^ | Retrospective; Single center | 2017-2019 | 314 (87/227) | NR | 38.6% vs. 36.3% (P>0.05) | 32.1% vs 31.0% (P>0.05) | 50.0% vs. 52.7% (P>0.05) | 3-year DFS 80.9% vs. 85.6% (P=0.322)  3-year OS 87.9% vs. 92.9% (P=0.258) | 3-year DFS 83.6% vs. 86.4% (P=0.819)  3-year OS 88.2% vs. 93.5% (P=0.633) | 3-year DFS 76.5% vs. 83.5% (P=0.319)  3-year OS 82.5% vs. 87.8% (P=0.200) | NR | NR | NR |
| Kang, 2022^6^ | Retrospective; Single center | 2014-2018 | 1572 (818/754) | In accordance with 2018 ASCO/CAP guidelines | 14.79% vs. 9.81% (P=0.003) | 5.43% vs 6.74% (P=0.4) | 26.82% vs. 22.6% (P=0.3) | 5-year DFS 71.6% vs. 77.8% (P=0.002)  5-year OS 84.1% vs. 92.4% (P<0.001) | 5-year DFS NS (P=0.66)  5-year OS NS (P=0.21) | 5-year DFS 65.6% vs. 76.4% (P=0.02)  5-year OS 75.4% vs. 83.8% (P=0.052) | NR | NR | NR |
| Domergue, 2022^7^ | Retrospective; Single center | 2005-2020 | 437 (316/121) | NR | NR | NR | 41.8% vs. 35.7% (P=0.284) | NR | NR | 5-year iDFS 65.4% vs. 60.6% (P=0.487)  5-year DDFS 68.5% vs. 63.1% (P=0.210)  5-year OS 72.9% vs. 70.0% (P=0.329) | NR | NR | NR |
| Miglietta, 2022^8^ | Retrospective; Single center | 2002-2018 | 446 (116/145) | Evaluated according to ASCO/CAP recommendations at the time of diagnosis, cases diagnosed before 2014 were reviewed to comply with the currently adopted 10% cutoff of HER2 IHC 3+ | 33.6% vs. 21.4% (P=0.035) | 12.1% vs 8.3% (P=0.721) | 42.2% vs. 34.2% (P=0.327) | DFS NS (P=0.27) | DFS NS (P=0.35) | DFS NS (P=0.79) | 26.4% | 8.9% | 14.8% |
| Shang, 2023^9^ | Retrospective; Single center | 2018-2021 | 1140 (164/429, Only report cases with residual disease) | NR | NR | NR | NR | NR | NR | NR | 21.42% | 6.46% | 10.19% |

Abbreviations: NR, not reported; HER2, human epidermal growth factor receptor-2; HR, ER, estrogen receptor; ratio; DFS, disease-free survival; iDFS, invasive disease-free survival; OS, overall survival; NAC, neoadjuvant chemotherapy; NS, no significance.

Reference:

1. Denkert C, Seither F, Schneeweiss A, Link T, Blohmer JU, Just M, et al. Clinical and molecular characteristics of HER2-low-positive breast cancer: pooled analysis of individual patient data from four prospective, neoadjuvant clinical trials. Lancet Oncol. 2021 Aug;22(8):1151-1161. doi: 10.1016/S1470-2045(21)00301-6. Epub 2021 Jul 9. PMID: 34252375.
2. de Moura Leite L, Cesca MG, Tavares MC, Santana DM, Saldanha EF, Guimarães PT, et al. HER2-low status and response to neoadjuvant chemotherapy in HER2 negative early breast cancer. Breast Cancer Res Treat. 2021 Nov;190(1):155-163. doi: 10.1007/s10549-021-06365-7. Epub 2021 Aug 18. PMID: 34409551.

3. Alves FR, Gil L, Vasconcelos de Matos L, Baleiras A, Vasques C, Neves MT, et al. Impact of Human Epidermal Growth Factor Receptor 2 (HER2) Low Status in Response to Neoadjuvant Chemotherapy in Early Breast Cancer. Cureus. 2022 Feb 17;14(2): e22330. doi: 10.7759/cureus.22330. PMID: 35371692; PMCID: PMC8938239.

4. Di Cosimo S, La Rocca E, Ljevar S, De Santis MC, Bini M, Cappelletti V, et al. Moving HER2-low breast cancer predictive and prognostic data from clinical trials into the real world. Front Mol Biosci. 2022 Sep 26; 9:996434. doi: 10.3389/fmolb.2022.996434. PMID: 36225259; PMCID: PMC9549400.

5. Shao Y, Yu Y, Luo Z, Guan H, Zhu F, He Y, et al. Clinical, Pathological Complete Response, and Prognosis Characteristics of HER2-Low Breast Cancer in the Neoadjuvant Chemotherapy Setting: A Retrospective Analysis. Ann Surg Oncol. 2022 Dec;29(13):8026-8034. doi: 10.1245/s10434-022-12369-4. Epub 2022 Aug 6. PMID: 35933542.

6. Kang S, Lee SH, Lee HJ, Jeong H, Jeong JH, Kim JE, et al. Pathological complete response, long-term outcomes, and recurrence patterns in HER2-low versus HER2-zero breast cancer after neoadjuvant chemotherapy. Eur J Cancer. 2022 Nov;176:30-40. doi: 10.1016/j.ejca.2022.08.031. Epub 2022 Sep 29. PMID: 36183652.

7. Domergue C, Martin E, Lemarié C, Jézéquel P, Frenel JS, Augereau P, et al. Impact of HER2 Status on Pathological Response after Neoadjuvant Chemotherapy in Early Triple-Negative Breast Cancer. Cancers (Basel). 2022 May 19;14(10):2509. doi: 10.3390/cancers14102509. PMID: 35626113; PMCID: PMC9139240.

8. Miglietta F, Griguolo G, Bottosso M, Giarratano T, Lo Mele M, Fassan M, et al. HER2-low-positive breast cancer: evolution from primary tumor to residual disease after neoadjuvant treatment. NPJ Breast Cancer. 2022 May 20;8(1):66. doi: 10.1038/s41523-022-00434-w. PMID: 35595761; PMCID: PMC9122970.

9. Shang J, Sun X, Xu Z, Cai L, Liu C, Wu S, et al. Evolution and clinical significance of HER2-low status after neoadjuvant therapy for breast cancer. Front Oncol. 2023 Feb 22;13:1086480. doi: 10.3389/fonc.2023.1086480. PMID: 36910643; PMCID: PMC9992719.
