# Supplementary material for: Pathological complete response, category change, and prognostic significance of HER2-low breast cancer receiving neoadjuvant treatment: a multicenter analysis of 2489 cases
Source: Br J Cancer. 2023 Aug 21;129(8):1274–83. doi: 10.1038/s41416-023-02403-x (PMC10575949; doi:10.1038/s41416-023-02403-x)
Supplement: Supplementary file 2 — Supplementary Figure [file 41416_2023_2403_MOESM2_ESM.pptx]

## Slide 1
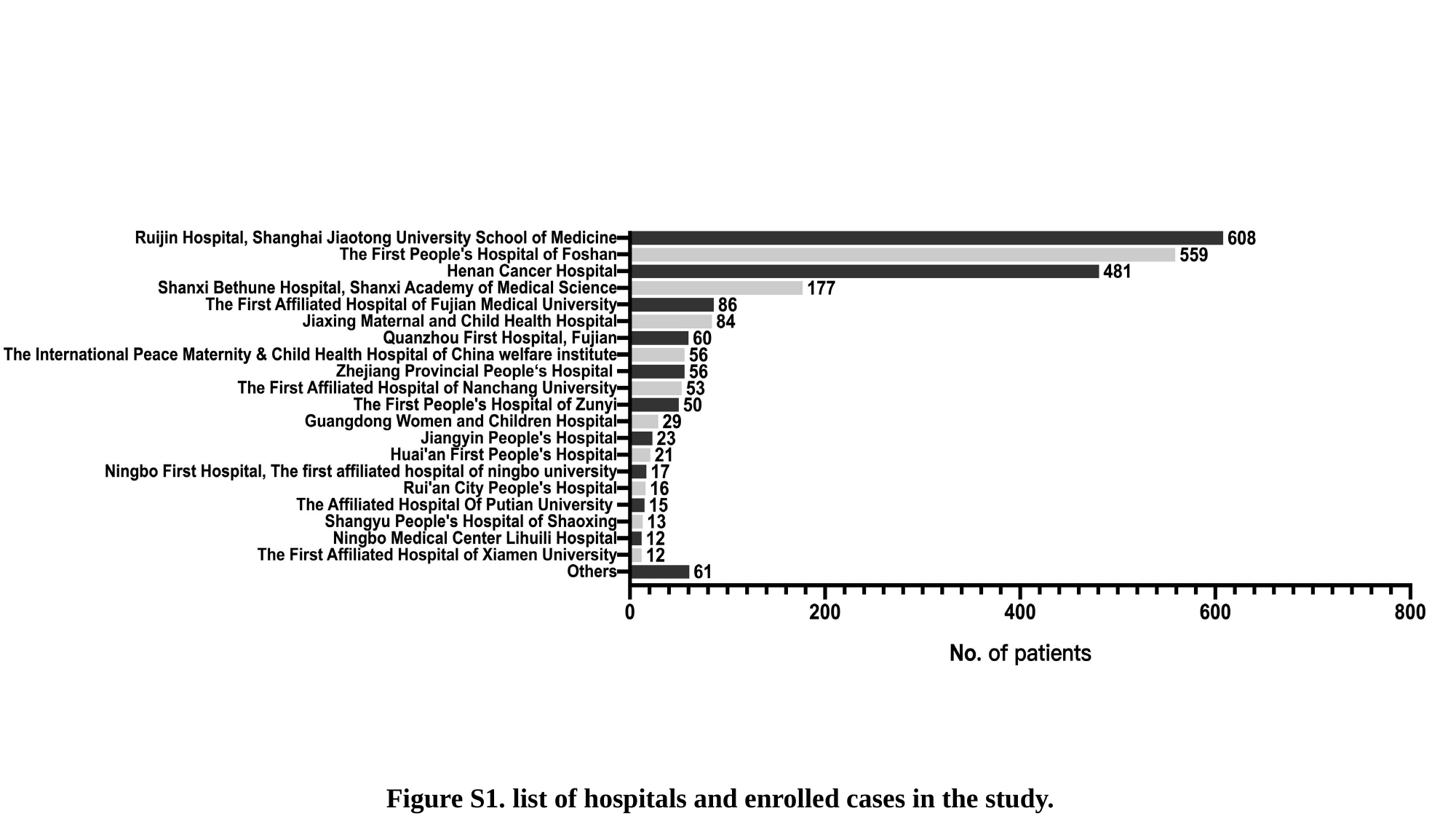

Figure S1. list of hospitals and enrolled cases in the study.

## Slide 2
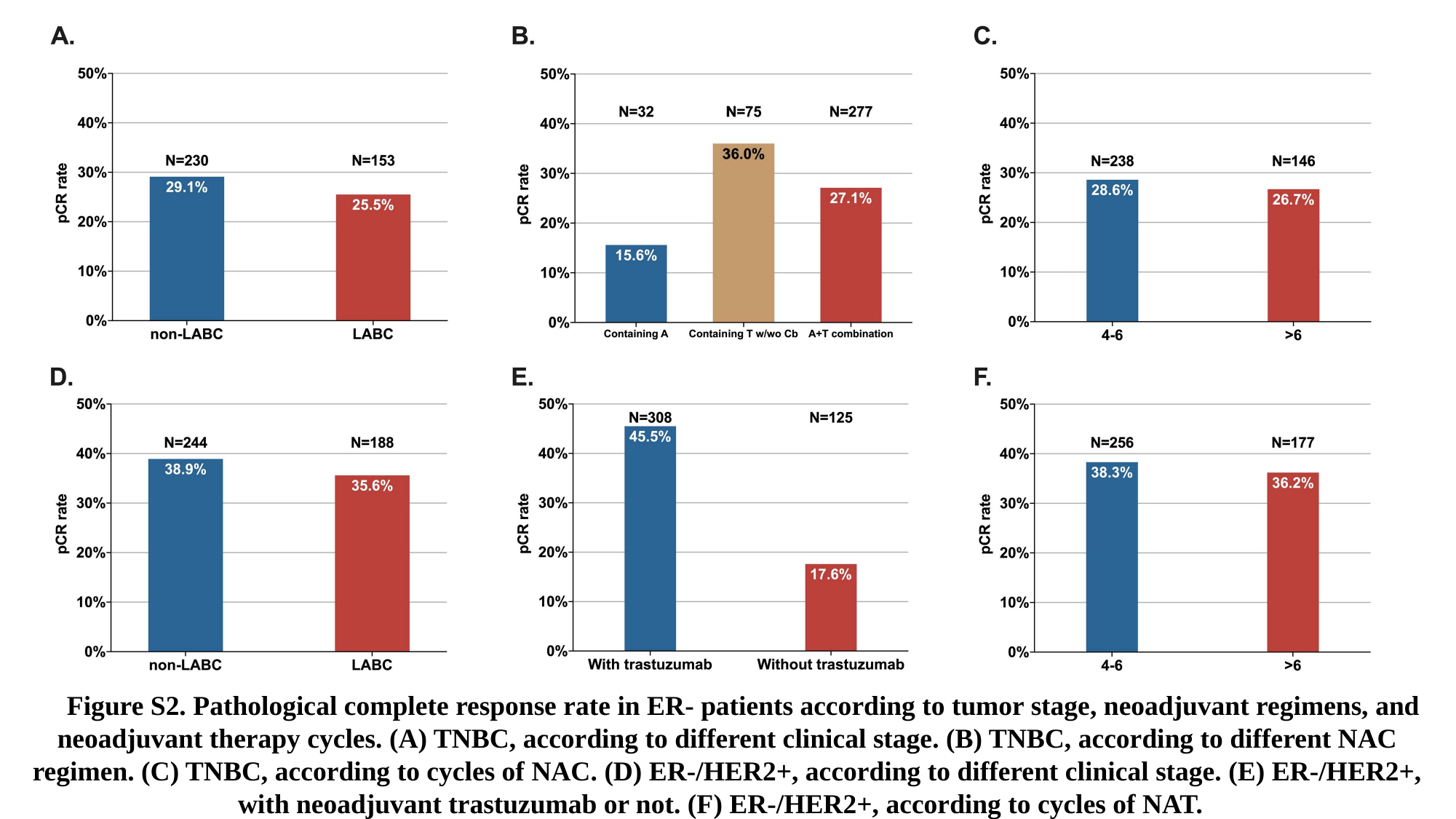

Figure S2. Pathological complete response rate in ER- patients according to tumor stage, neoadjuvant regimens, and neoadjuvant therapy cycles. (A) TNBC, according to different clinical stage. (B) TNBC, according to different NAC regimen. (C) TNBC, according to cycles of NAC. (D) ER-/HER2+, according to different clinical stage. (E) ER-/HER2+, with neoadjuvant trastuzumab or not. (F) ER-/HER2+, according to cycles of NAT.

## Slide 3
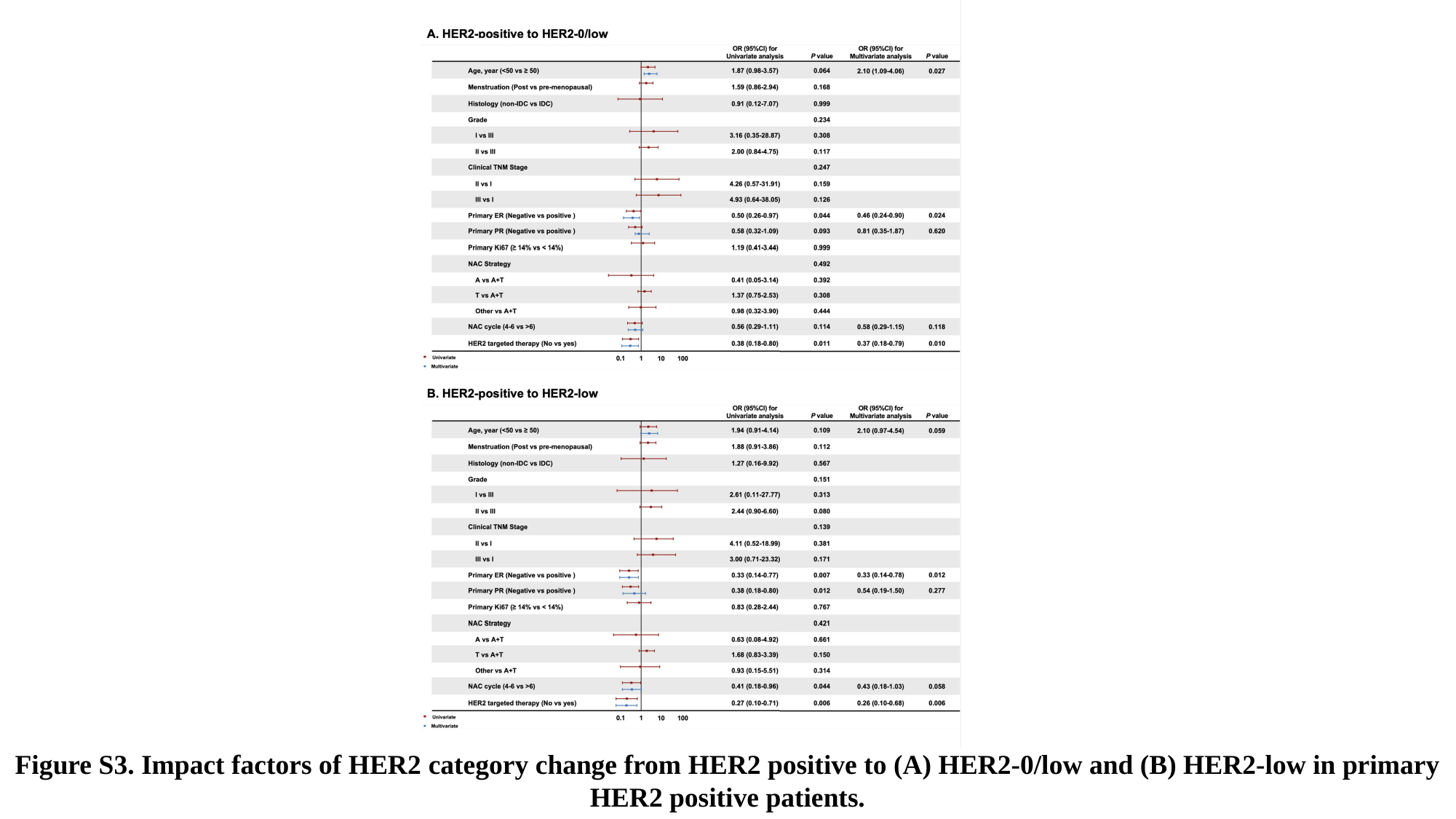

Figure S3. Impact factors of HER2 category change from HER2 positive to (A) HER2-0/low and (B) HER2-low in primary HER2 positive patients.

## Slide 4
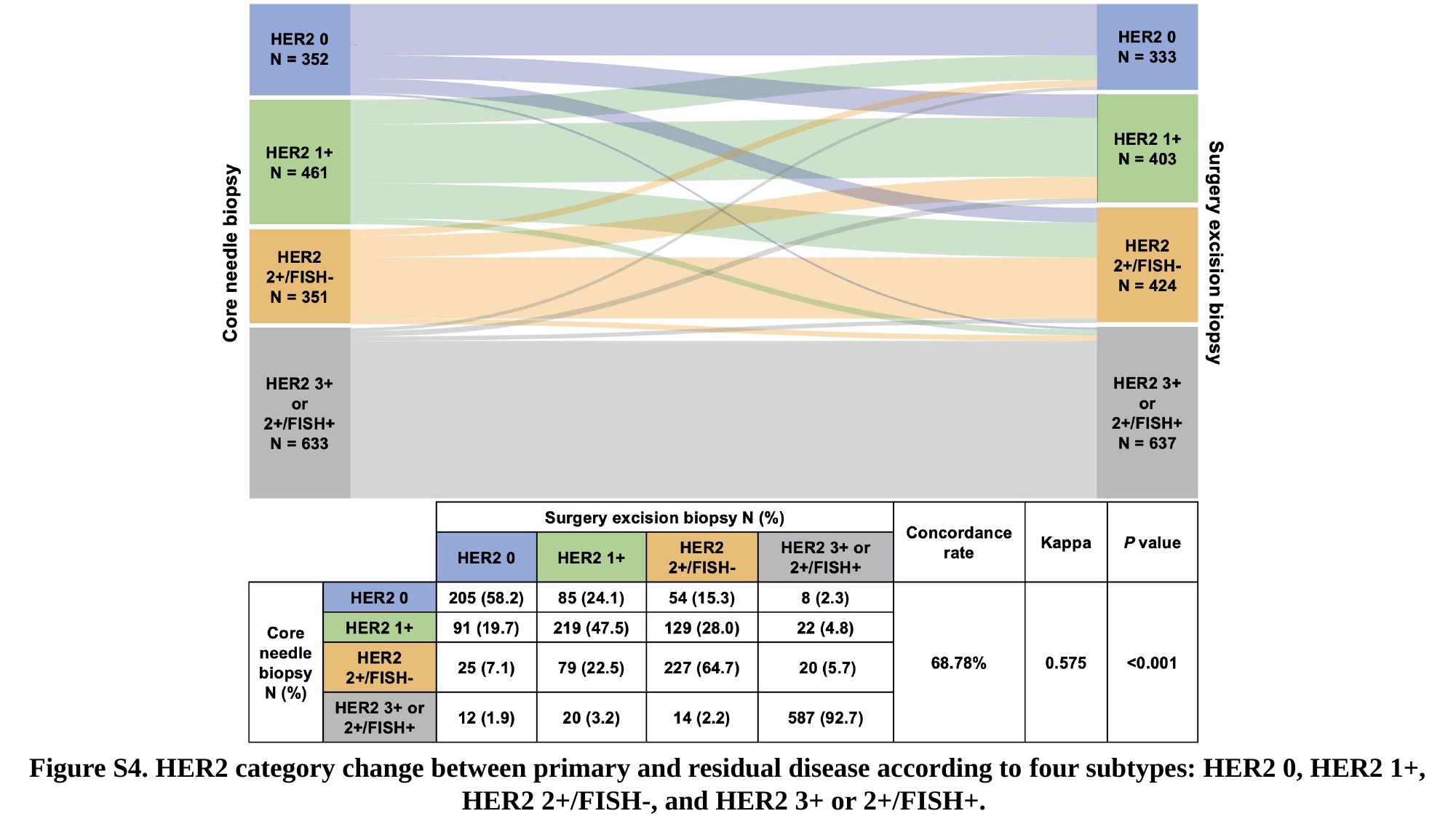

Figure S4. HER2 category change between primary and residual disease according to four subtypes: HER2 0, HER2 1+, HER2 2+/FISH-, and HER2 3+ or 2+/FISH+.

## Slide 5
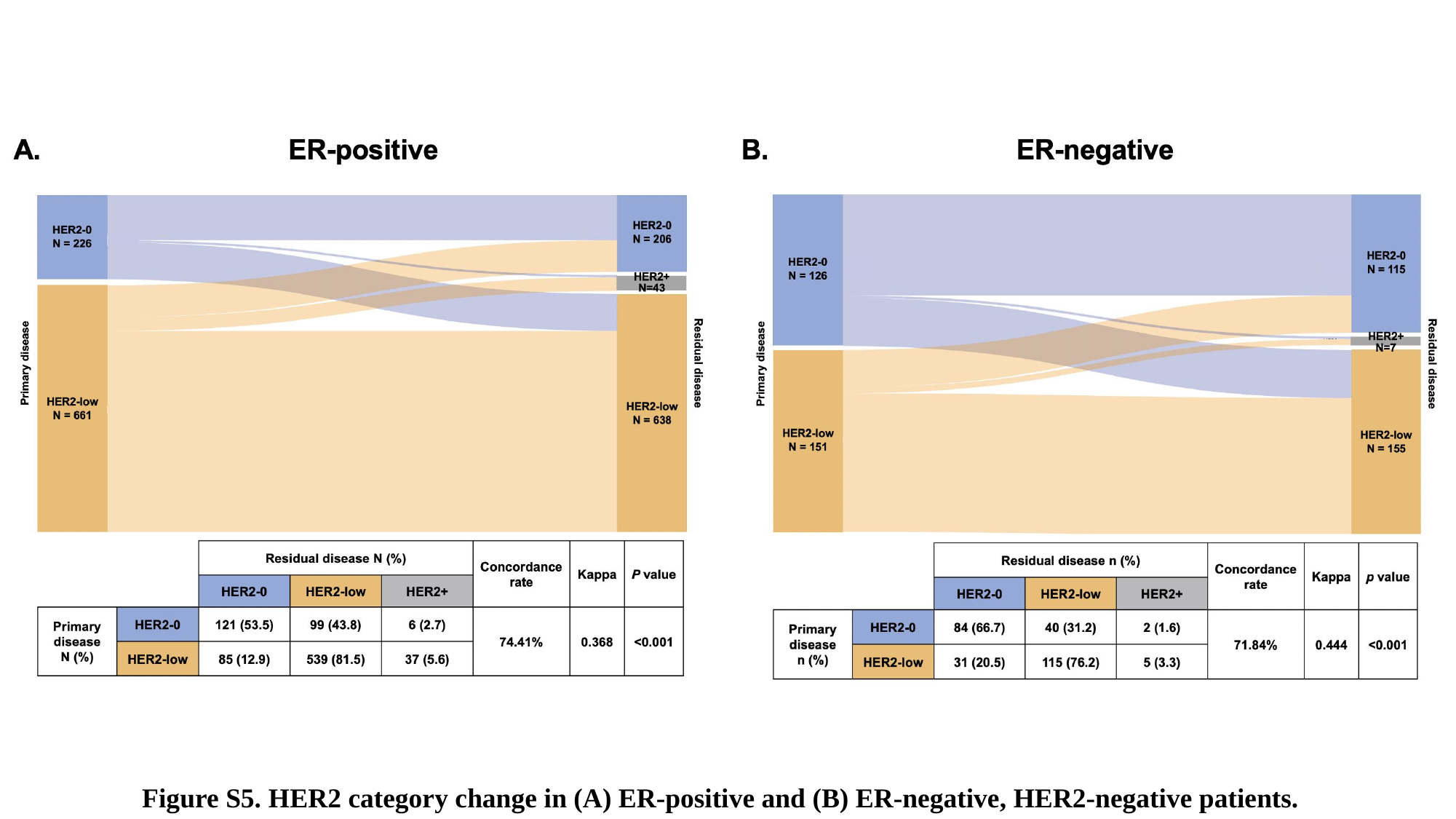

Figure S5. HER2 category change in (A) ER-positive and (B) ER-negative, HER2-negative patients.

## Slide 6
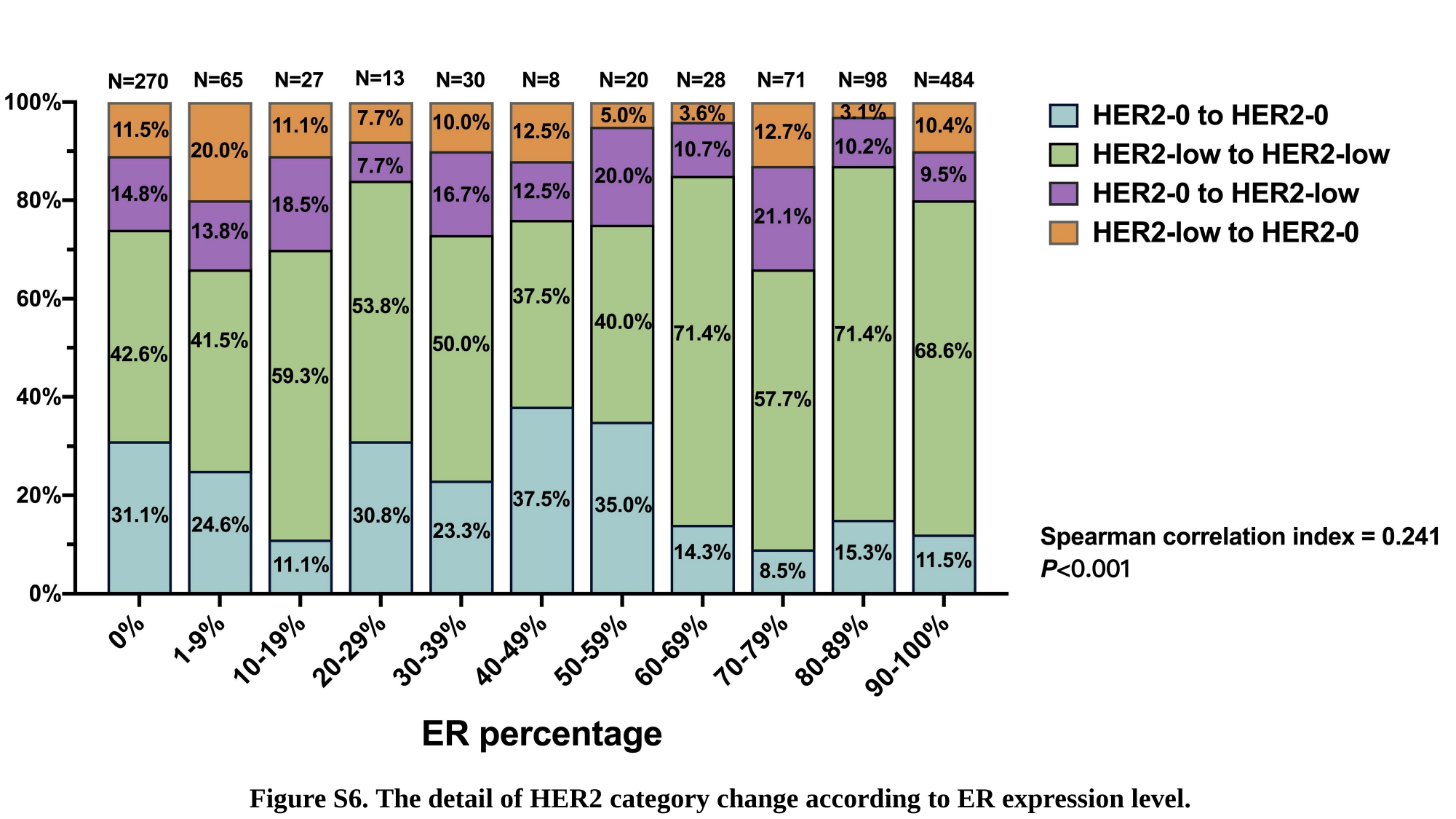

Figure S6. The detail of HER2 category change according to ER expression level.

## Slide 7
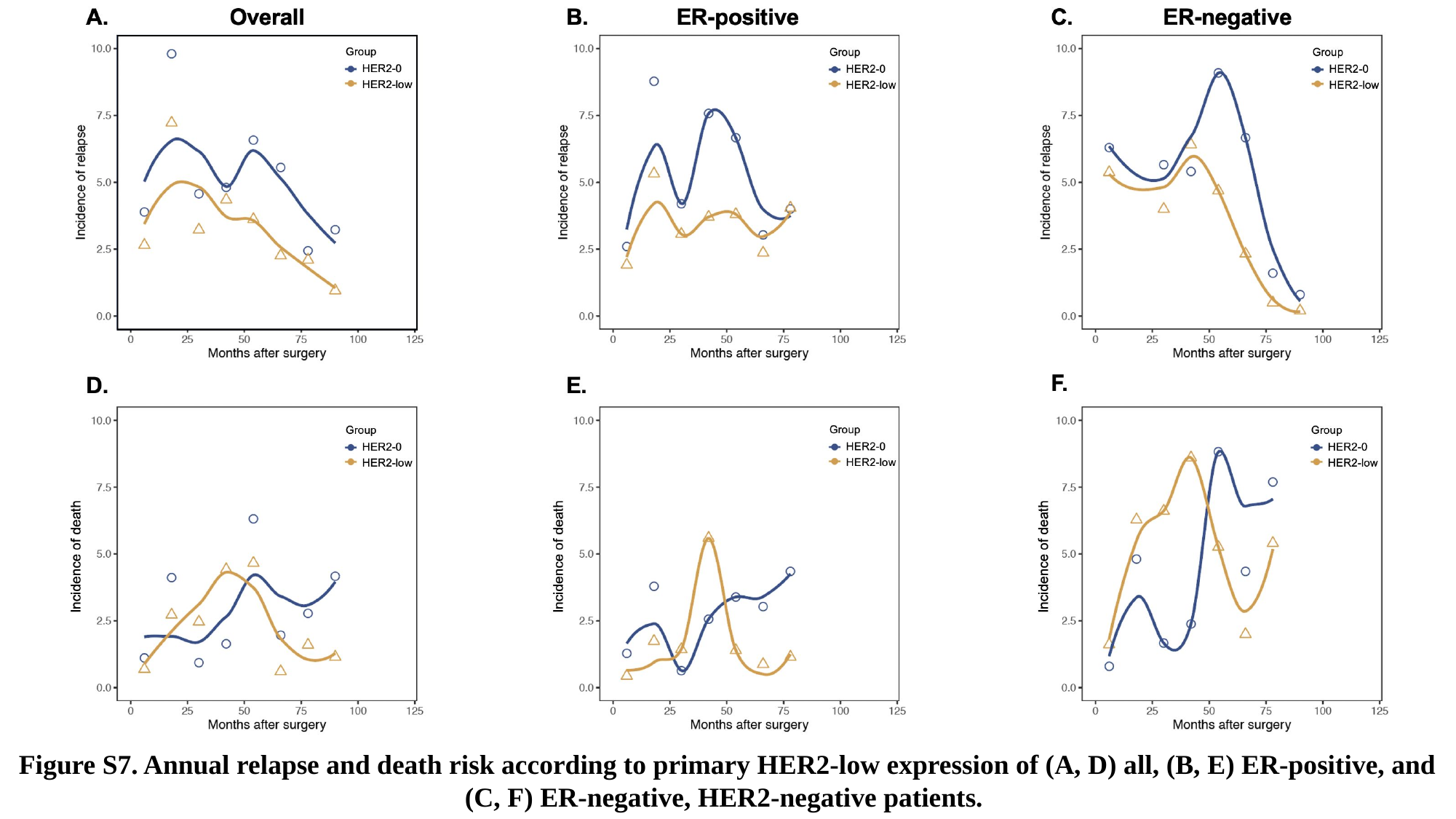

Figure S7. Annual relapse and death risk according to primary HER2-low expression of (A, D) all, (B, E) ER-positive, and (C, F) ER-negative, HER2-negative patients.

## Slide 8
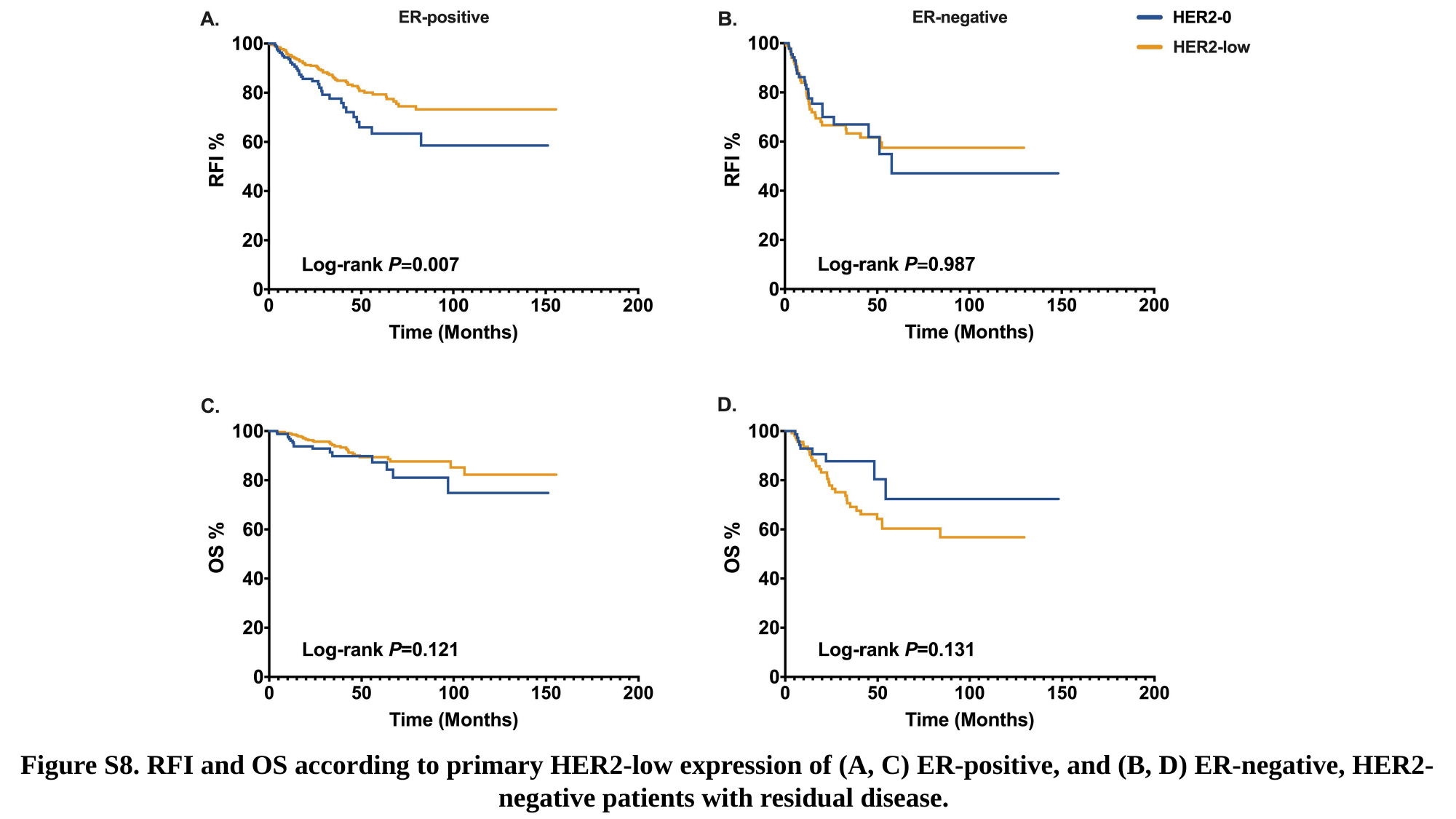

Figure S8. RFI and OS according to primary HER2-low expression of (A, C) ER-positive, and (B, D) ER-negative, HER2-negative patients with residual disease.

## Slide 9
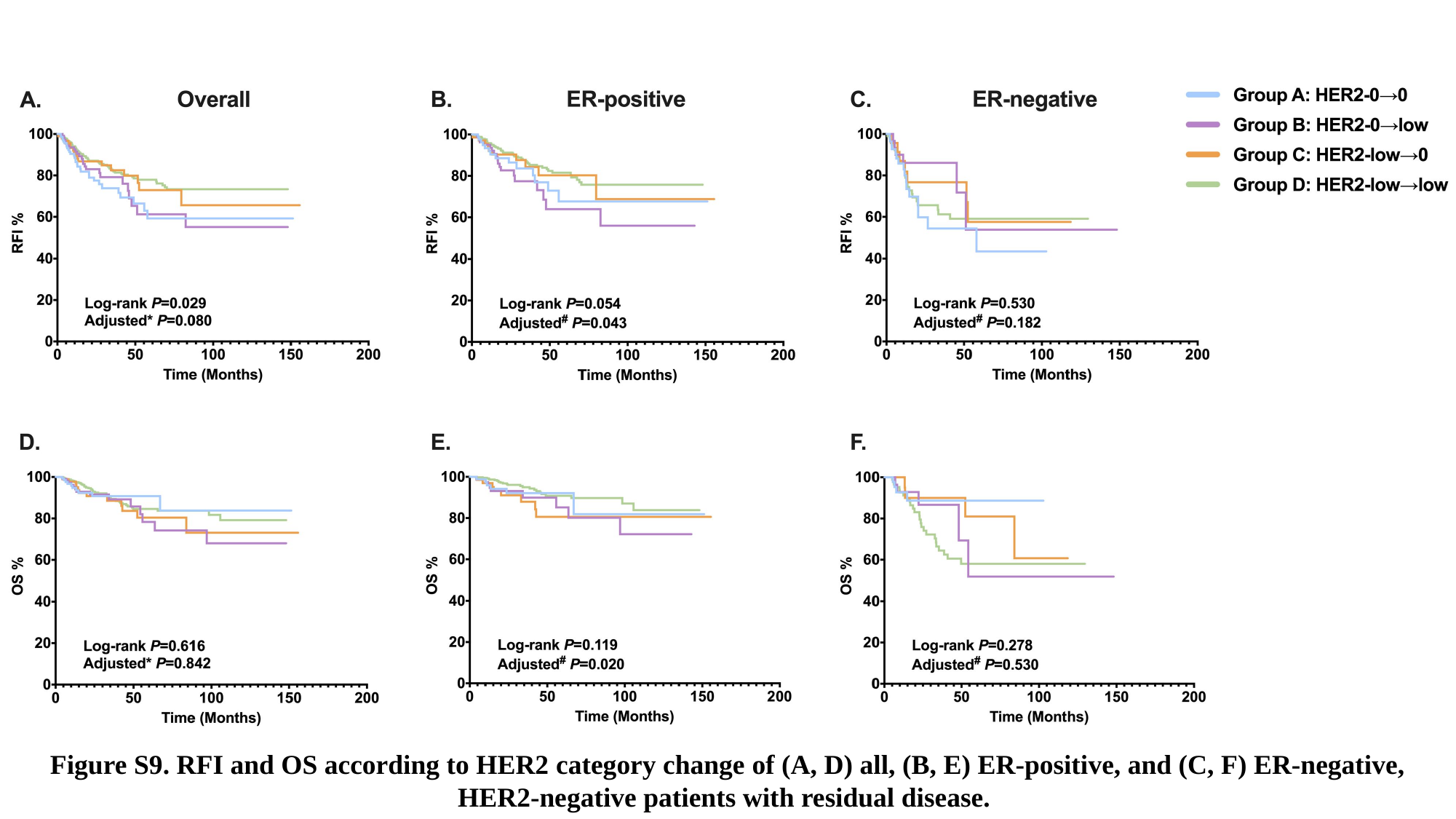

Figure S9. RFI and OS according to HER2 category change of (A, D) all, (B, E) ER-positive, and (C, F) ER-negative, HER2-negative patients with residual disease.

## Slide 10
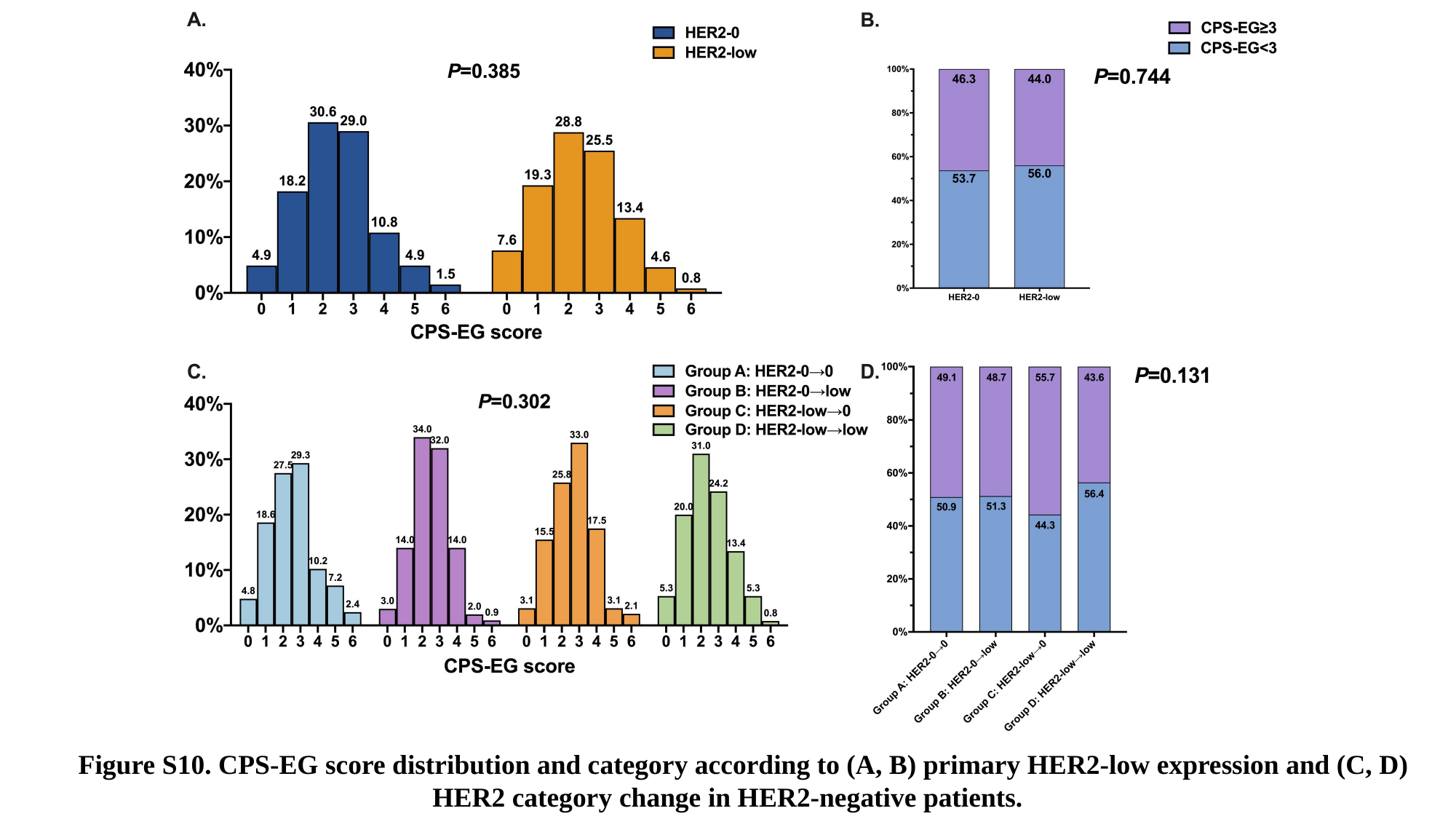

Figure S10. CPS-EG score distribution and category according to (A, B) primary HER2-low expression and (C, D) HER2 category change in HER2-negative patients.
